# Identification of a disulfidptosis-related lncRNA signature for the prognostic and immune landscape prediction in head and neck squamous cell carcinoma

**DOI:** 10.1007/s12672-024-00932-2

**Published:** 2024-03-14

**Authors:** Zhengyu Wei, Chongchang Zhou, Yi Fang, Hongxia Deng, Zhisen Shen

**Affiliations:** 1 Department of Otorhinolaryngology Head and Neck Surgery, The Affiliated Lihuili Hospital, Ningbo University, Ningbo, Zhejiang China; 2 Department of Otorhinolaryngology Head and Neck Surgery, Ningbo Medical Center Lihuili Hospital, Ningbo, Zhejiang China; 3 Health Science Center, Ningbo University, Ningbo, Zhejiang China

**Keywords:** Disulfidptosis, Immunotherapy, Head and neck squamous cell carcinoma, Prognosis, Long noncoding RNA

## Abstract

**Purpose:**

Disulfidptosis, a newly identified form of cell death, is triggered by disulfide stress. Herein, a unique signature was developed based on disulfidptosis-related lncRNAs (DRlncRNAs) for the prognostic and immune landscape prediction of head and neck squamous cell carcinoma (HNSCC).

**Methods:**

Transcriptome, somatic mutation, and clinical data were acquired at The Cancer Genome Atlas database. Individuals were partitioned into training and test cohorts at a 1:1 ratio to facilitate the development of a DRlncRNA signature using the least absolute shrinkage and selection operation method. Based on the median risk score, all HNSCC individuals were stratified into the high-risk group (HRG) and low-risk group (LRG). Kaplan–Meier survival and time-dependent receiver operating characteristic (ROC) analyses were used to estimate the prognostic value, and a nomogram was generated for survival prediction. To provide a more comprehensive assessment, the tumor microenvironment, functional enrichment, immune cell infiltration, and immunotherapeutic sensitivity were explored between LRG and HRG.

**Results:**

A DRlncRNA signature was established with 10 DRlncRNAs. The corresponding values of areas under the ROC curves for 1–, 3–, and 5–year overall survival were 0.710, 0.692, and 0.640. A more favorable prognosis was noted in the patients with lower risk, along with higher immune scores, increased immune-related functions, and immune cell infiltration, as well as improved response to the immunotherapeutic intervention in comparison with individuals at higher risk.

**Conclusion:**

These findings demonstrate that the developed DRlncRNA signature holds promise as a reliable prognostic marker and predictor of immunotherapy response in HNSCC patients.

**Supplementary Information:**

The online version contains supplementary material available at 10.1007/s12672-024-00932-2.

## Introduction

Head and neck squamous cell carcinoma (HNSCC) is the sixth most prevalent malignancy worldwide, with around 600,000 new cases of HNSCC diagnosed around the world annually [1–3]. HNSCC is a highly aggressive tumor arising from the oral cavity, oropharynx, hypopharynx, and larynx [4, 5]. Alcoholism, smoking, and infection with human papillomavirus are recognized as common risk factors [5]. Early diagnosis of HNSCC is challenging due to its concealed physiological location, resulting in the majority of patients being diagnosed at advanced stages [5]. Patients with advanced HNSCC have an unfavorable prognosis, irrespective of receiving interventions such as surgery, radiotherapy, and chemotherapy [6]. Immunotherapy, including immune checkpoint inhibitors (ICIs), is a promising strategy to treat HNSCC [7]. However, the overall response rate of immunotherapy in unselected HNSCC patients falls below 20% [8, 9]. Thus, it is crucial to investigate new biomarkers and establish innovative prognostic signatures for predicting the outcomes of individuals with HNSCC and enabling personalized precision therapy.

Disulfidptosis, a recently identified cell death form, is induced by disulfide stress, as reported in recent research [10]. Liu et al. noted a novel type of cell death termed disulfidoptosis, which exhibited substantial differences from apoptosis and ferroptosis. They reported that elevated expression of SLC7A11 in combination with glucose starvation is the underlying cause of this biological process [10]. In cells with high SLC7A11 expression under glucose starvation, high cystine uptake combined with the decreased NADPH supply results in the depletion of NADPH, aberrant disulfide bonding in actin cytoskeleton proteins, collapse of the actin filament network, eventually leading to disulfidptosis [10, 11]. In addition, Rac activated the WAVE regulatory complex to promote lamellipodia formation, which facilitated disulfidptosis [12]. It was found that SLC7A11, SLC3A2, RPN1, and NCKAP1 were the top four disulfidptosis-promoting genes [11]. Recent studies reported the association between disulfidptosis-related genes (DRGs) and prognosis in thyroid carcinoma, hepatocellular carcinoma, and bladder cancer [13–15]. Nevertheless, despite the available literature, the relationship between disulfidptosis and HNSCC remains obscure.

Long noncoding RNAs (lncRNAs), a form of essential regulators involved in diverse biological processes, assume a significant role in tumorigenesis, such as proliferation, apoptosis, and metastasis [16]. According to previous studies, lncRNAs have been considered functionally active in the progression and development of HNSCC [17, 18] and hold promise as novel biomarkers and therapeutic strategies for HNSCC [19, 20]. Nonetheless, the association between lncRNAs and disulfidptosis in HNSCC is still largely unexplored. Hence, in this study, disulfidptosis-related lncRNAs (DRlncRNAs) were retrieved at The Cancer Genome Atlas (TCGA, https://portal.gdc.cancer.gov/) [21] database using Pearson correlation analysis. Afterward, utilizing a combined approach of Cox proportional hazard regression and Least Absolute Shrinkage and Selection Operator (LASSO) regression, a DRlncRNA signature for HNSCC was developed based on 10 prognostic lncRNAs. Moreover, the signature was examined further concerning its prognostic value. Further investigations were conducted to assess its relevance in various aspects, such as immunotherapy response, infiltration of immune cells, and tumor microenvironment (TME).

## Methods

### HNSCC datasets and clinical data retrieval

The gene expression datasets of HNSCC, along with comprehensive clinical information, were acquired from the TCGA database (last accessed: July 15, 2023). The current research involved a total of 44 adjacent normal control cases and 522 HNSCC cases. The transcriptome data were retrieved in the form of fragments per kilobase million. The corresponding clinicopathologic information was also extracted, encompassing age, sex, clinical stage, grade, as well as T-, N-, and M-categories, along with overall survival (OS) time and status. Detailed clinicopathologic information of HNSCC patients used in this study was shown in Supplementary Table 1.

### Expression data of DRlncRNAs in HNSCC

An assessment of prior research resulted in acquiring a total of 10 DRGs, including OXSM, GYS1, LRPPRC, NDUFS1, NUBPL, RPN1, NCKAP1, NDUFA11, SLC3A2, and SLC7A11 [10, 22]. Through the Pearson correlation analysis, DRlncRNAs were selected employing the "limma" R packages. This analysis was executed after preparing 10 DRGs and all lncRNA expression data from the TCGA-HNSCC dataset. The lncRNAs were established as being linked to 10 DRGs: |Correlation coefficient|> 0.4 and *p* < 0.001. The screened lncRNAs were then classified as DRlncRNAs.

### Construction of the DRlncRNA-based prognostic model

Cases with corresponding prognostic information in the entire dataset were randomly categorized into training and test cohorts at a 1:1 ratio for further assessment. Screening of the survival-related DRlncRNAs in the training cohort was conducted through univariate Cox regression at a value of *p* < 0.05. Afterward, LASSO regression with tenfold cross-validation and a *p*-value of 0.05 was applied to further refine the selection of DRlncRNAs for constructing the DRlncRNA-based prognostic model. To prevent overfitting, this process was repeated 1000 times. The final selection of DRlncRNAs was conducted on the basis of multivariate Cox regression analysis. For all HNSCC patients, the formula mentioned below was utilized for quantifying the risk score:

$$\mathrm{Risk\, score} = {\sum }_{\mathrm{i }= 1}^{{\text{n}}}{\text{coefficient}} \times \mathrm{DRlncRNA\, expression}$$

The median risk score in the training set was employed for categorizing the training and test cohorts into high-risk group (HRG) and low-risk group (LRG). The R packages employed in this process encompassed "survival", "caret", "glmnet", "survminer", and "timeROC".

### Validation of the risk model

To examine potential correlations between the calculated risk score and survival, we generated plots illustrating the risk score distribution of individuals with HNSCC. The Kaplan–Meier (KM) curves of OS and progression-free survival (PFS) were utilized to illustrate the predictive performance of the risk signature. The log-rank test was employed to compare the difference in survival between HRG and LRG. Moreover, heatmaps were utilized for the visualization of the expression of DRlncRNAs in each cohort. These analyses utilized R packages including "survival", "survminer", and "pheatmap". The next step involved utilizing principal component analysis (PCA) to show the distinguish of all genes, DRGs, DRlncRNAs, and risk model lncRNAs. The analysis was conducted utilizing the R packages "limma" and "scatterplot3d". Following this, the independent predictive value of the risk scores for OS was examined through Cox regression analyses (both univariate and multivariate). To comprehensively investigate the effectiveness of the DRlncRNA model, the receiver operating characteristic (ROC) curves of 1–, 3–, and 5–years were generated. Further assessment of the predictive performance of the risk score was executed through a comparison with clinical information (age, sex, grade, and stage) using ROC curves and C-index. These clinical features were also used for subgroup survival analyses. Additionally, the DRlncRNA signature was assessed to determine its potential for superior predictive performance. This was accomplished by comparing time-dependent ROC curves and C-index values with four previously established signatures [23–26]. The R packages "survival", "survminer", "timeROC", "dplyr", "rms", and "pec" were employed for this assessment.

### Tumor mutation burden (TMB) analysis

In the waterfall plot, the top 15 genes with the highest mutation frequencies were shown for both HRG and LRG. An analysis was then conducted to determine the mutation counts of all the HNSCC samples. The KM plotter and log-rank test were performed to test the OS of subgroups based on risk score and TMB utilizing the R packages "survival" and "survminer".

### Establishment and validation of the prognostic nomogram

A nomogram was developed for survival prediction considering such factors as age, sex, histologic grade, clinical stage, T–, M–, and N–categories and risk score. Time-dependent ROC curves and calibration curves were employed to assess the precision of the nomogram. The R packages used for these analyses included "survival", "regplot", "rms", "survcomp", "survminer", and "timeROC".

### Analysis of the association between the risk model and clinical characteristics

To validate the clinical relevance of the developed signature, the correlation of the risk score with clinicopathological variables was assessed. These variables encompassed clinical stages, sex, age, grade, T–, and N–categories. The Wilcoxon signed-rank test was applied to detect variance in risk scores for diverse groups of clinicopathological features. The R packages "ComplexHeatmap", "limma", and "ggpubr" were used to perform the necessary statistical analysis.

### Functional enrichment analysis

To identify differentially expressed genes (DEGs) between two groups, the "limma" R tool with a significance threshold of FDR q < 0.05 and |log2FC|≥ 1 was utilized. Next, the identified genes were utilized for gene ontology (GO) annotation and KEGG pathway enrichment analysis.

### Immune landscape and immunotherapy prediction analyses

To gain further insights into the immune landscape of the process, various analyses were conducted utilizing the "ESTIMATE" package, such as StromalScore, ImmuneScore, Tumor purity, and ESTIMATEScore for individuals with HNSCC. Subsequently, the immune functions of the two groups were compared using single-sample gene set enrichment analysis (ssGSEA). The CIBERSORT method was utilized to compute the percentages of 22 types of immune cells in every HNSCC specimen to determine the potential relationship between the risk score and the infiltration of immune cells. Further, the Tumor Immune Dysfunction and Exclusion (TIDE, http://tide.dfci.harvard.edu/) scores were adopted to predict the response of HNSCC cases to immunotherapy. Conventionally, a lower TIDE score reflects, a more favorable response to immunotherapy. Moreover, to further predict the immunotherapy response of patients with HNSCC, the expression of human leukocyte antigen (HLA)-related and ICIs-related genes in HRG and LRG was comparatively assessed. The R packages utilized for this investigation were "ggpubr", "plyr", "reshape2", "ggplot2", and "ggExtra".

### Statistical analyses

The analyses were carried out using R version 4.2.2, which also serves as a data visualization tool. The Wilcoxon signed-rank test or Chi-square test was employed for comparing the variables. Pearson correlation analysis was utilized for correlation evaluations. *P* value < 0.05 was deemed as statistically significant.

## Results

### Expression analysis of DRlncRNAs in HNSCC patients and construction of the DRlncRNA risk model

**Fig. 1:**
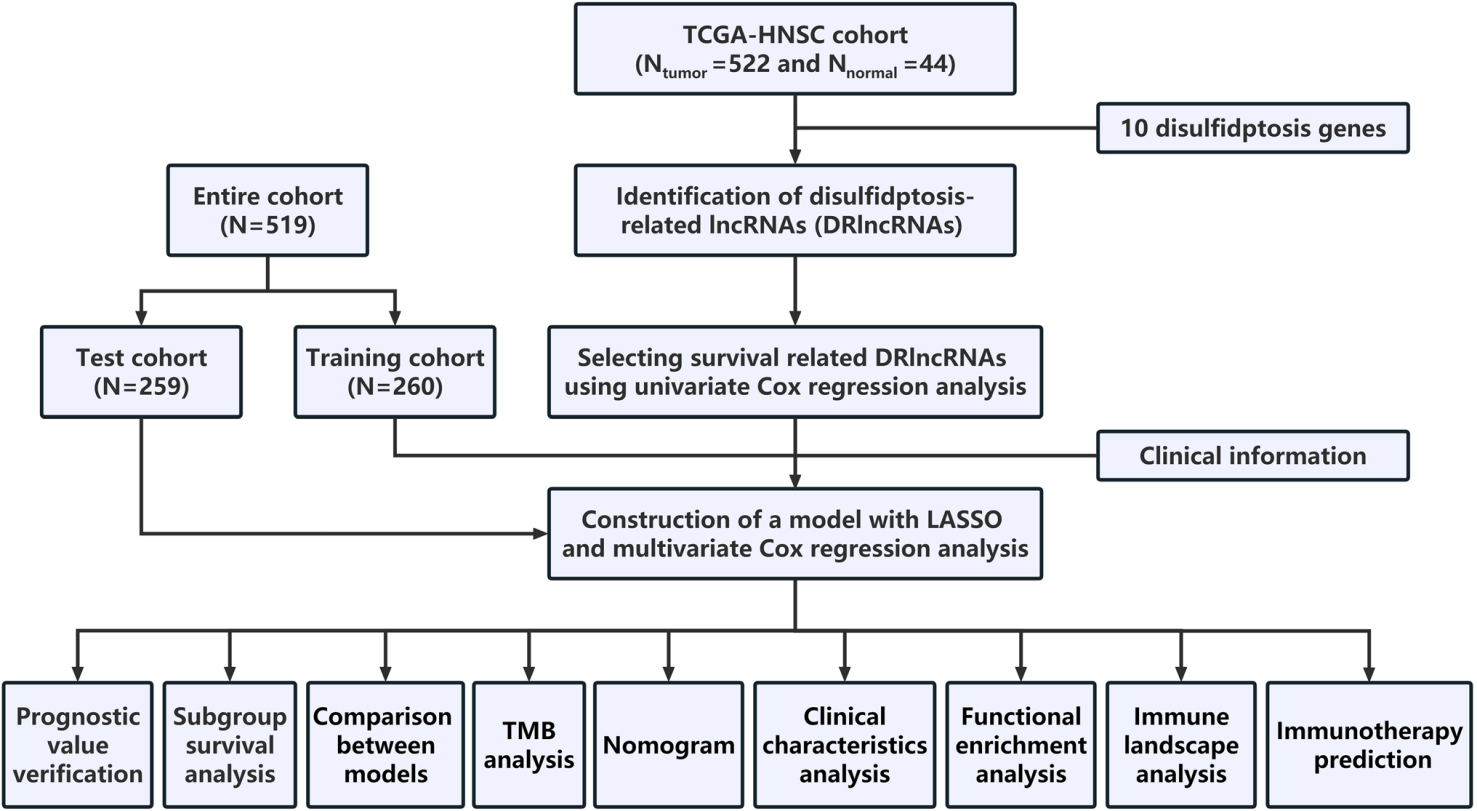
Flowchart of the study design. *TCGA* the Cancer Genome Atlas, *LASSO* Least Absolute Shrinkage and Selection Operator, *TMB* tumor mutation burden

**Fig. 2:**
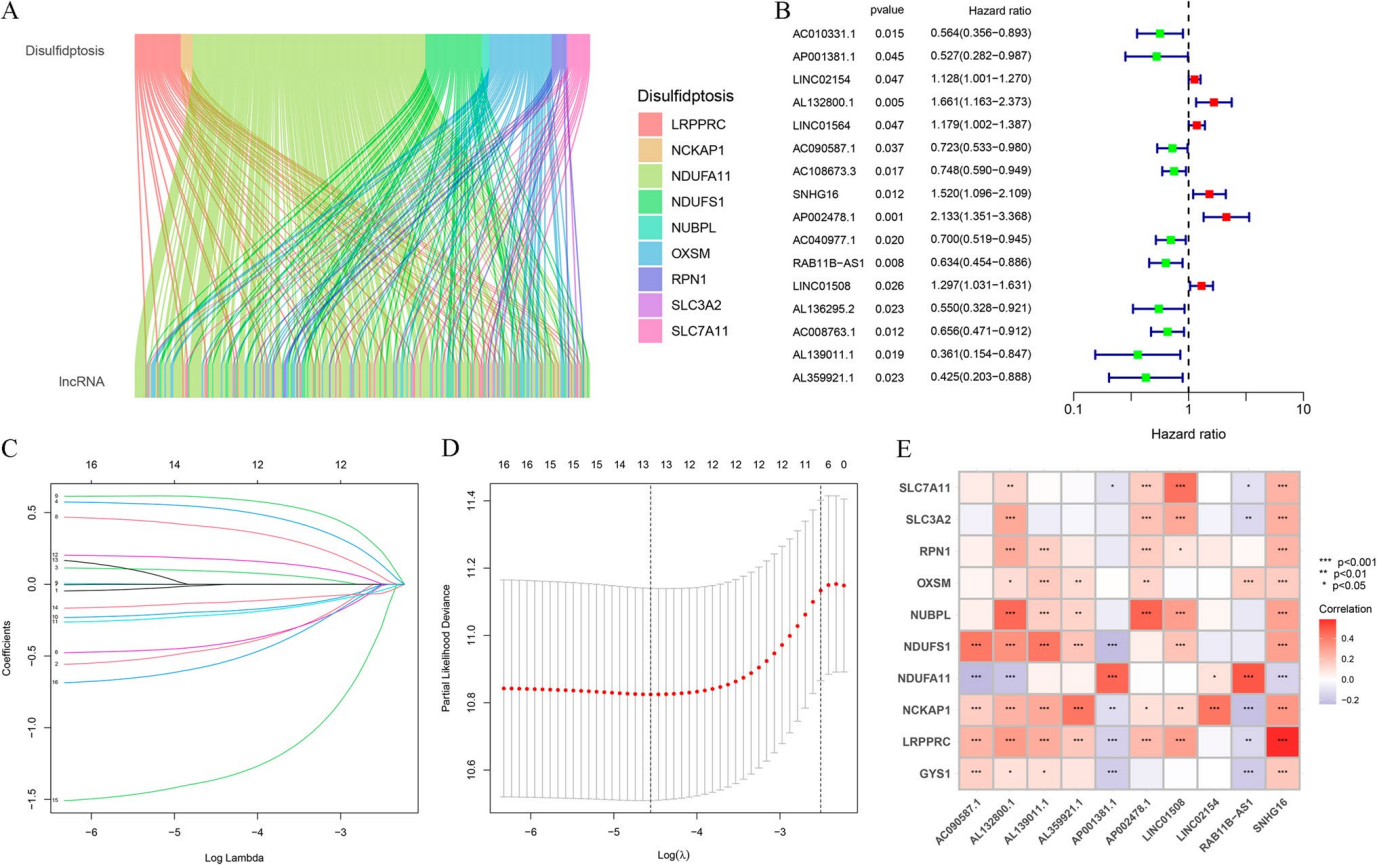
Expression analysis of disulfidptosis-related lncRNAs (DRlncRNAs) in TCGA-HNSCC dataset and establishment of a DRlncRNAs prognostic signature. **A** Co-expression analysis of disulfidptosis-related genes (DRGs) and lncRNAs. **B** Univariate Cox regression analysis based prognostic value of DRlncRNAs (*P* < 0.05). **C** Diagrammatic representation of LASSO expression coefficients. **D** Cross-validation plot for the penalty term of LASSO analysis. **E** Correlation heatmap showing the expression correlation between ten lncRNAs included in the model and the DRGs

The study flow is illustrated in Fig. 1. Following the acquisition of the data from the TCGA-HNSCC dataset, consisting of 44 normal and 522 HNSCC samples, we identified 292 DRlncRNAs through the co-expression analysis (Fig. 2A, Supplementary Table 2), which involved comparing the expression patterns of lncRNAs from the TCGA dataset and a list of DRGs obtained from previous research [10, 22]. Subsequently, individuals with HNSCC in the TCGA dataset, along with corresponding prognostic information, underwent random separation into training (260 patients) and test cohorts (259 patients). Table 1 provides a summary of the clinicopathological features of these individuals. No notable differences were observed in these features between the two cohorts. Univariate Cox regression analyses were applied to examine the predictive value of DRlncRNAs, culminating in the selection of 16 DRlncRNAs linked to survival (Fig. 2B, P < 0.05). Of these, 6 were identified as prognostic factors contributing to worse prognoses for patients with HNSCC (hazard ratio, HR > 1), while the remaining DRlncRNAs were found to reduce the risk of disease for these individuals. A prognostic model was then constructed based on these DRlncRNAs using multivariate and LASSO Cox regression analyses (Fig. 2C, D). The correlation analysis between DRGs and lncRNAs included in the risk model is shown in Fig. 2E. It was found that lncRNA AL132800.1 and SNHG16 exhibited positive correlations with the majority of DRGs, with the exception of NDUFA11. The given formula was employed to calculate the risk scores of individuals with HNSCC: $\mathrm{Risk score}\hspace{0.17em}=\hspace{0.17em}AP001381.1\hspace{0.17em}\times \hspace{0.17em}(- 0.587273043296351)\hspace{0.17em}+\hspace{0.17em}LINC02154\hspace{0.17em}\times \hspace{0.17em}0.112318861447426\hspace{0.17em}+\hspace{0.17em}AL132800.1\hspace{0.17em}\times \hspace{0.17em}0.581499235156066\hspace{0.17em}+\hspace{0.17em}AC090587.1\hspace{0.17em}\times \hspace{0.17em}(- 0.459482287352876)\hspace{0.17em}+\hspace{0.17em}SNHG16\hspace{0.17em}\times \hspace{0.17em}0.476331659786032\hspace{0.17em}+\hspace{0.17em}AP002478.1\hspace{0.17em}\times \hspace{0.17em}0.590399134093677\hspace{0.17em}+\hspace{0.17em}RAB11B-AS1\hspace{0.17em}\times \hspace{0.17em}(- 0.366444716506785)\hspace{0.17em}+\hspace{0.17em}LINC01508\hspace{0.17em}\times \hspace{0.17em}0.209030440721399\hspace{0.17em}+\hspace{0.17em}AL139011.1\hspace{0.17em}\times \hspace{0.17em}(- 1.71114221248055)\hspace{0.17em}+\hspace{0.17em}AL359921.1\hspace{0.17em}\times \hspace{0.17em}(- 0.894430728855104)$.

**Table 1:** Clinical characteristics of the patients with head and neck squamous carcinomas in this study

Covariates	Type	Training	Test	Total	P value
Age	< = 65	175 (67.31%)	166 (64.09%)	341 (65.7%)	0.4971
	> 65	85 (32.69%)	93 (35.91%)	178 (34.3%)	
Sex	Female	73 (28.08%)	63 (24.32%)	136 (26.2%)	0.3831
	Male	187 (71.92%)	196 (75.68%)	383 (73.8%)	
Histologic grade	G1	35 (13.46%)	27 (10.42%)	62 (11.95%)	0.4607
	G2	155 (59.62%)	148 (57.14%)	303 (58.38%)	
	G3	60 (23.08%)	65 (25.1%)	125 (24.08%)	
	G4	2 (0.77%)	5 (1.93%)	7 (1.35%)	
	Unknow	8 (3.08%)	14 (5.41%)	22 (4.24%)	
Clinical stage	Stage I	17 (6.54%)	10 (3.86%)	27 (5.2%)	0.2974
	Stage II	36 (13.85%)	34 (13.13%)	70 (13.49%)	
	Stage III	44 (16.92%)	37 (14.29%)	81 (15.61%)	
	Stage IV	124 (47.69%)	142 (54.83%)	266 (51.25%)	
	Unknow	39 (15%)	36 (13.9%)	75 (14.45%)	
T category	T0	0 (0%)	1 (0.39%)	1 (0.19%)	0.4249
	T1	28 (10.77%)	20 (7.72%)	48 (9.25%)	
	T2	71 (27.31%)	64 (24.71%)	135 (26.01%)	
	T3	45 (17.31%)	54 (20.85%)	99 (19.08%)	
	T4	83 (31.92%)	91 (35.14%)	174 (33.53%)	
	Unknow	33 (12.69%)	29 (11.2%)	62 (11.95%)	
M category	M0	94 (36.15%)	91 (35.14%)	185 (35.65%)	0.9914
	M1	0 (0%)	1 (0.39%)	1 (0.19%)	
	Unknow	166 (63.85%)	167 (64.48%)	333 (64.16%)	
N category	N0	88 (33.85%)	87 (33.59%)	175 (33.72%)	0.9907
	N1	33 (12.69%)	34 (13.13%)	67 (12.91%)	
	N2	82 (31.54%)	87 (33.59%)	169 (32.56%)	
	N3	4 (1.54%)	4 (1.54%)	8 (1.54%)	
	Unknow	53 (20.38%)	47 (18.15%)	100 (19.27%)	

### Survival analysis of HNSCC patients based on DRlncRNAs signature score

**Fig. 3:**
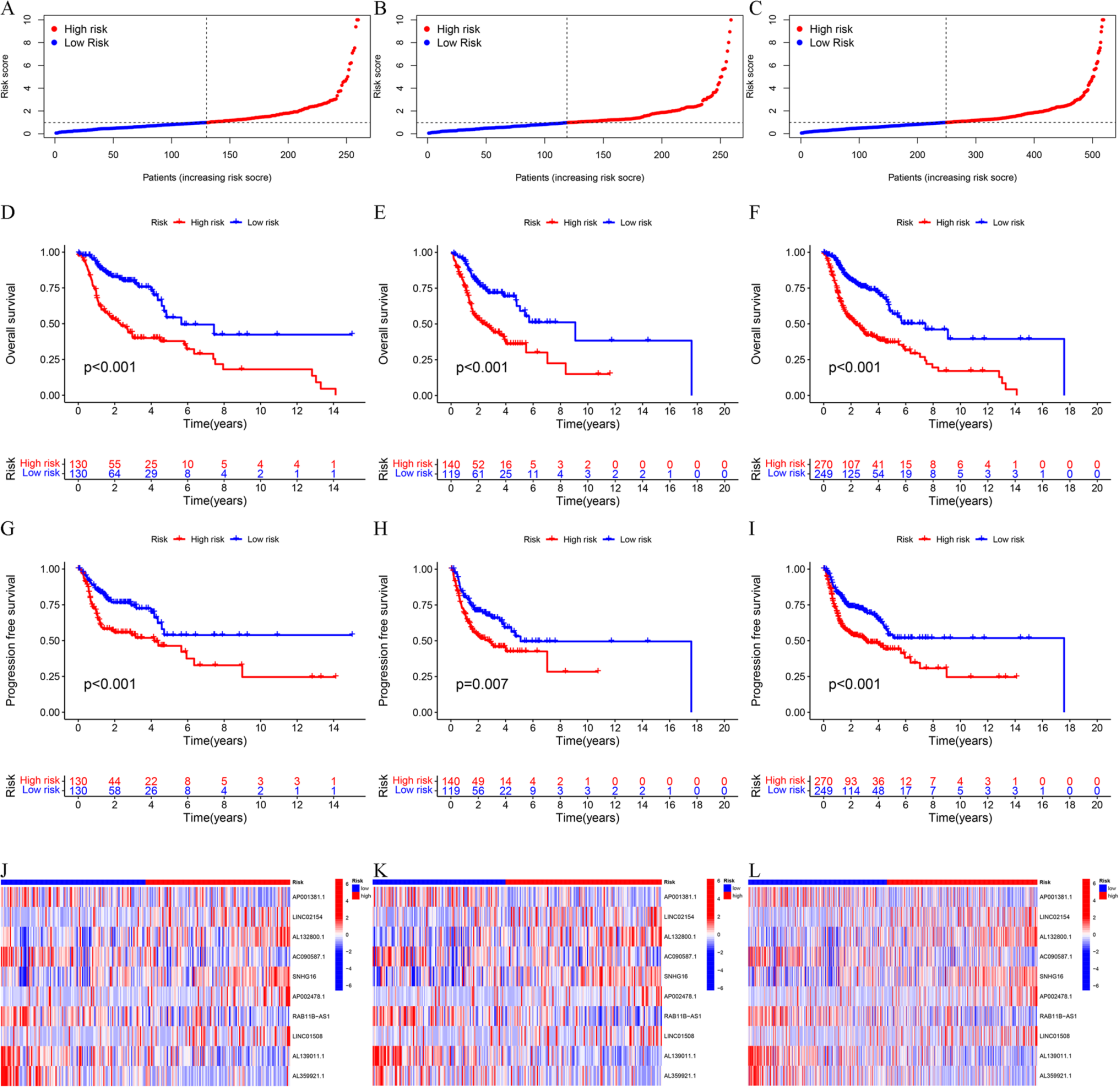
Survival analysis of head and neck squamous cell carcinoma (HNSCC) patients based on DRlncRNAs signature score. Distributions of risk scores of individuals with HNSCC in training cohort (**A**), test cohort (**B**), and entire TCGA-HNSCC dataset (**C**). Kaplan–Meier (KM) curves of overall survival (OS) in training (**D**), test cohorts (**E**), and the entire TCGA-HNSCC dataset (**F**). KM curves of progression-free survival in training (**G**), test cohorts (**H**), and the entire TCGA-HNSCC dataset (**I**). Heatmaps of DRlncRNAs expression in training cohort (**J**), test cohort (**K**), and entire TCGA-HNSCC dataset (**L**)

Based on the median risk score, individuals with HNSCC were categorized into HRG and LRG. The distributions of risk scores (Fig. 3A) in the training cohort revealed an increase in the death rates increased with higher risk scores. Similar patterns were observed in the test cohort (Fig. 3B) and the entire TCGA-HNSCC cohort (Fig. 3C). Moreover, the KM curves of OS in the training (Fig. 3D), test (Fig. 3E), and entire TCGA-HNSCC cohorts (Fig. 3F) implied an unfavorable prognosis for the individuals in the HRG than the LRG counterparts (*P* < 0.001). Similarly, the KM curves of PFS in the training (Fig. 3G, P < 0.001), test (Fig. 3H, P = 0.007), and entire TCGA-HNSCC cohorts (Fig. 3I, P < 0.001) indicated the same trend. Additionally, heatmaps (Fig. 3J–L) suggested that the levels of DRlncRNAs LINC02154, AL132800.1, SNHG16, AP002478.1, and LINC01508 were mainly elevated in the HRG, whereas elevated expression of AP001381.1, AC090587.1, RAB11B-AS1, AL139011.1, and AL359921.1 was noted in the LRG. The above findings revealed that our prognostic risk model divided HNSCC patients into HRG and LRG, and patients in the LRG may had better OS than those in the HRG, suggesting that the risk score was negatively correlated with OS.

### Prognosis value of the DRlncRNA-based risk model

**Fig. 4:**
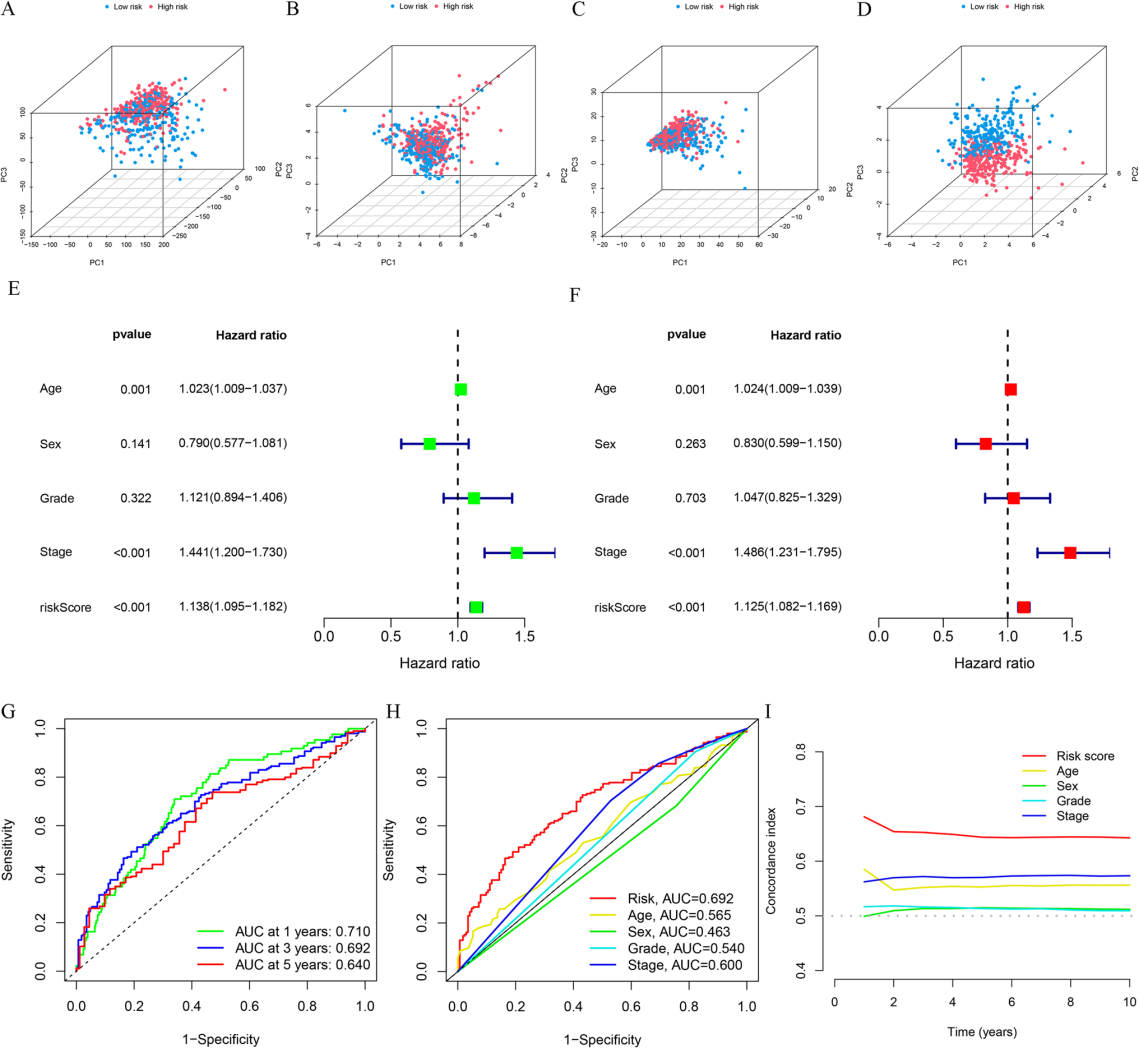
Prognosis value of the DRlncRNAs signature. Principal component analysis to show the distinguish of all genes (**A**), DRGs (**B**), DRlncRNAs (**C**), and risk model lncRNAs (**D**). Demonstration of the independent prognostic nature of risk score through univariate (**E**) and multivariate (**F**) analyses in the individuals with HNSCC. **G** The receiver operating characteristic (ROC) curve of the DRlncRNAs signature for 1–, 3–, and 5–years OS. **H** ROC curve of the risk score and diverse clinicopathological parameters (age, sex, histologic grade, and clinical stage). **I** C-index value of the risk score and other clinicopathological variables

**Fig. 5:**
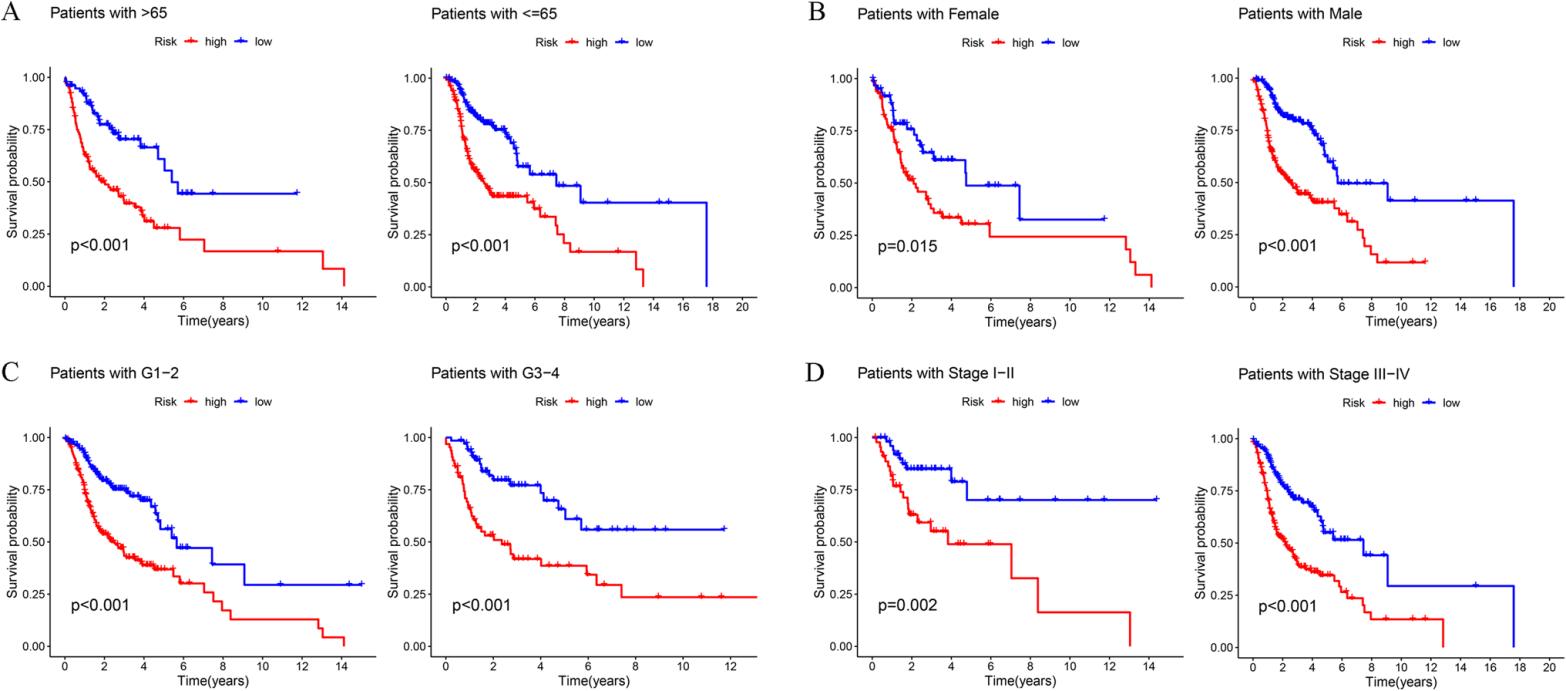
Subgroup survival analysis. Kaplan–Meier survival curves for age (**A**), sex (**B**), histologic grade (**C**), and clinical stage (**D**) in the TCGA-HNSCC dataset

**Fig. 6:**
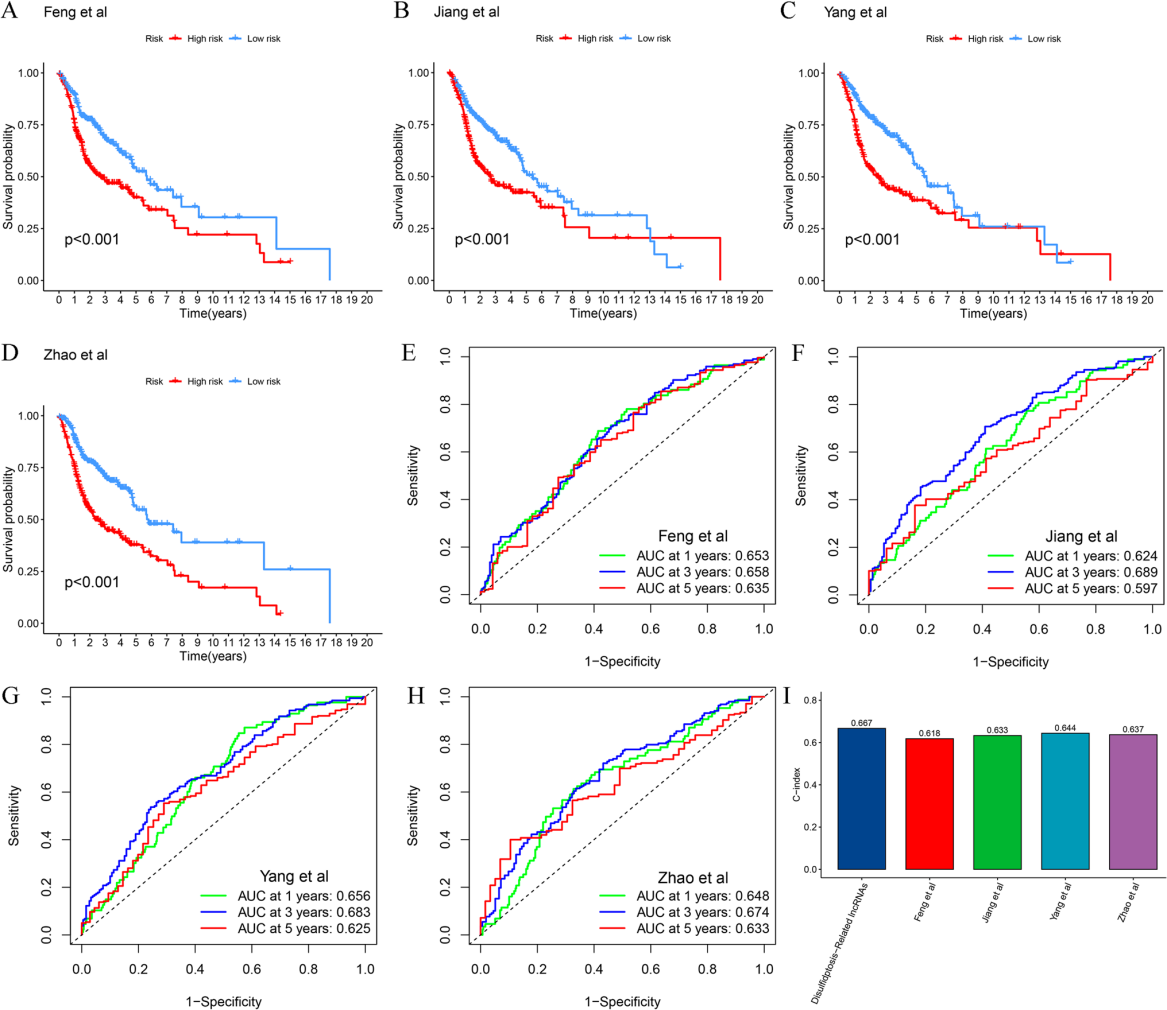
Comparative evaluation of the performance of the DRlncRNAs and previously published signatures in the TCGA-HNSCC dataset. Kaplan–Meier survival analysis of Feng (**A**), Jiang (**B**), Yang (**C**), and Zhao signatures (**D**). Time-dependent ROC curves of Feng (**E**), Jiang (**F**), Yang (**G**), and Zhao signatures (**H**). **I** C-index for all signatures

The results of the PCA indicated that the DRlncRNA signature (Fig. 4D) could be differentiated based on the risk status of HNSCC patients compared to the all genes (Fig. 4A), DRGs (Fig. 4B), DRlncRNAs (Fig. 4C). Univariate (Fig. 4E) and multivariate (Fig. 4F) analyses further confirmed that the risk score of the DRlncRNA-based risk model, age, and clinical stage could function as independent risk factors influencing the prognosis of individuals with HNSCC. Subsequently, the area under the ROC curve (AUC) values for 1–, 3–, and 5–year OS were calculated to be 0.710, 0.692, and 0.640, respectively, for the entire TCGA-HNSCC cohort (Fig. 4G). Furthermore, the ROC curve (Fig. 4H) and C-index (Fig. 4I) demonstrated that the risk score had a better predictive performance for HNSCC compared to various clinicopathological features, such as age, sex, histologic grade, and clinical stage. These features were also considered in conducting a subgroup analysis. The analysis of different clinical subgroups revealed that individuals in the LRG depicted a more favorable survival prognosis across all clinical subgroups, including age (Fig. 5A), sex (Fig. 5B), histologic grade (Fig. 5C), and clinical stage (Fig. 5D). Further, in order to assess the performance of the DRlncRNAs signature, KM curves were plotted, and the AUC values and C-index were determined for four previously published signatures were calculated in the TCGA-HNSCC cohort (Fig. 6). All four of these signatures effectively stratified the individuals into two subgroups with notably different prognostic outcomes (Fig. 6A–D). However, our DRlncRNAs signature outperformed these four signatures (Fig. 6E–H) in terms of the AUC values for 1–, 3–, and 5–year OS. Notably, our DRlncRNAs signature depicted the highest C-index value of 0.667 among the signatures (Fig. 6I). These results showed that the risk score model had a good capacity in OS prediction.

### TMB analysis

**Fig. 7:**
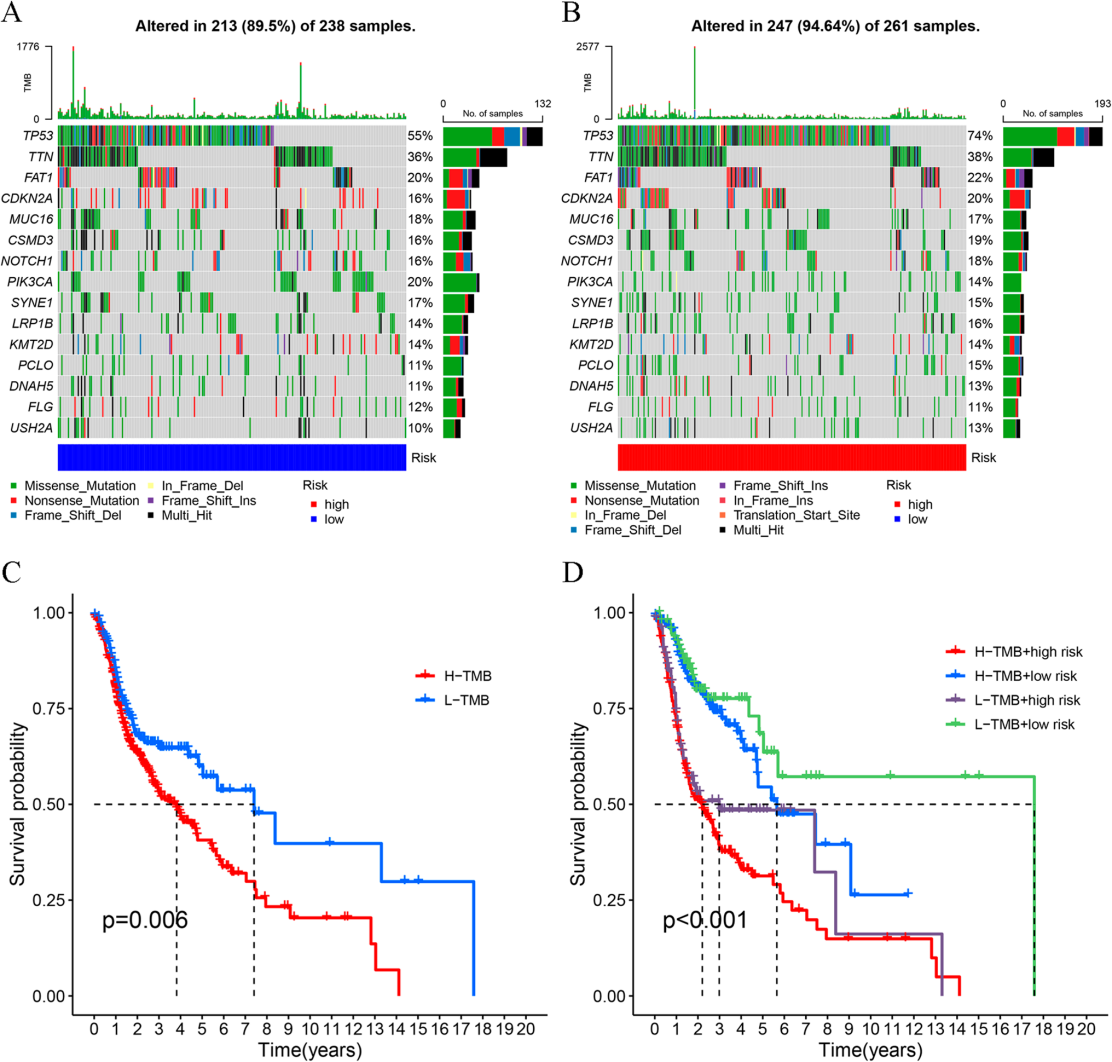
TMB analysis. **A** Waterfall plots illustrating the top 15 mutated genes in the low-risk group. **B** Waterfall plots visualizing the mutations in the high-risk group. **C** Survival analysis between the two TMB cohorts (high and low). **D** Survival analysis incorporating TMB and risk scores for individuals with HNSCC

A comparison of the first 15 mutated genes in individuals from the LRG and HRG divided by their risk scores is shown in Fig. 7A and B. This comparison was based on the available somatic mutation information. Considering the significance of the TMB in clinical practice, it was quantified for each sample. The resulting data implied that the survival time of individuals with high TMB was shorter in comparison with those with low TMB (Fig. 7C, P = 0.006). As shown by the stratified subgroup survival analysis, individuals with elevated risk scores had a poorer OS in comparison with those with lower risk scores in both TMB groups (Fig. 7D, P < 0.001).

### Establishment and validation of the DRlncRNAs risk model-based Nomogram

**Fig. 8:**
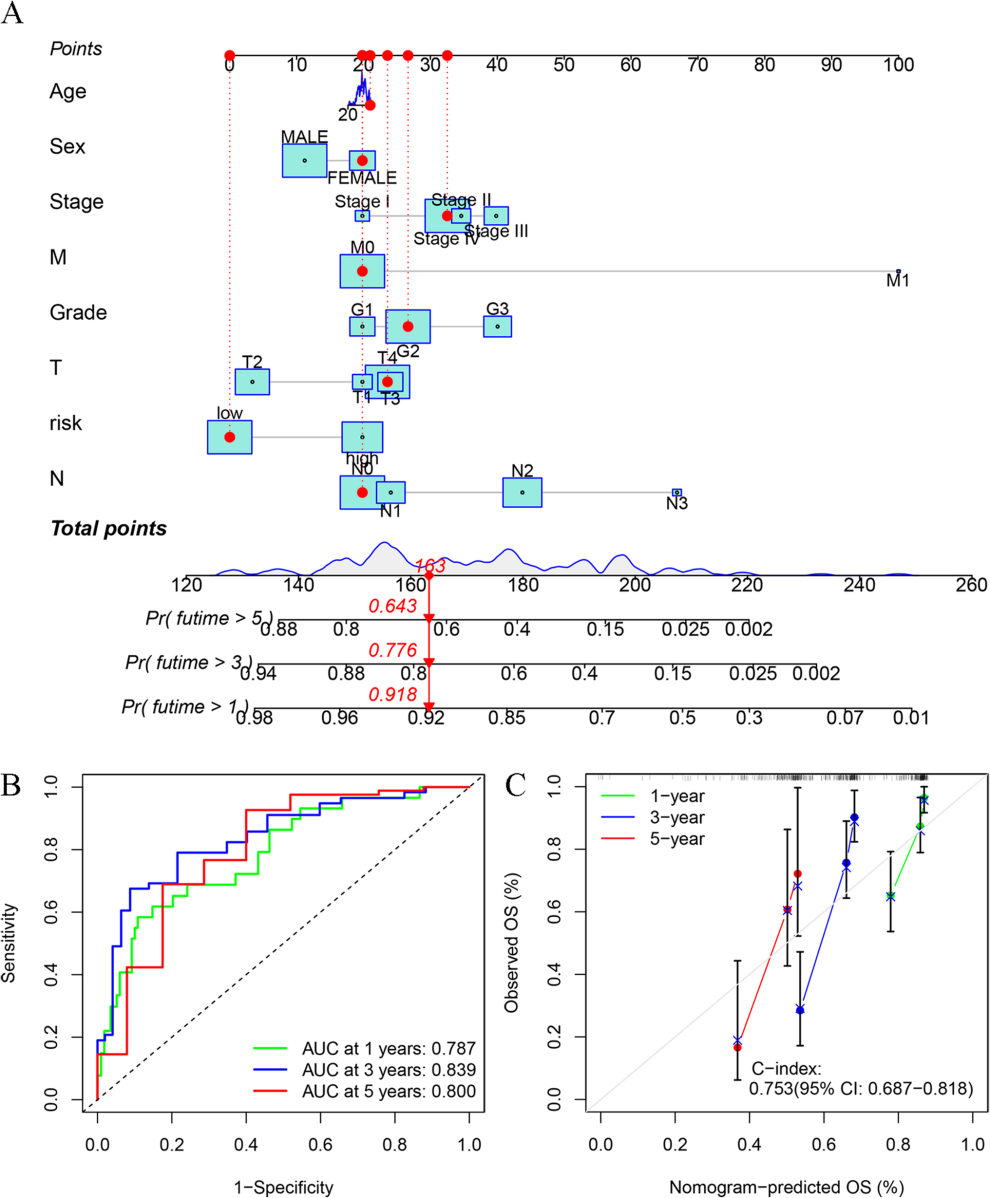
Development of a nomogram. **A** Nomogram for prediction of the 1–, 3–, and 5–year prognosis based on clinicopathological variables and risk scores. **B** Time-dependent ROC curves of the nomogram. (**C**) Calibration curves plot of the nomogram

A nomogram was developed for prediction of 1–, 3–, and 5–year OS for individuals with HNSCC (Fig. 8A). To examine the prediction performance of the nomogram, the ROC and calibration curves were generated. As shown in Fig. 8B, the corresponding AUC values of the nomogram for 1–, 3–, and 5–year OS were 0.787, 0.839, and 0.800, respectively. Furthermore, the calibration curves for the nomogram (Fig. 8C) showed close alignment with the theoretical line, and the value of the C-index was 0.753 (95% CI 0.687 − 0.818), indicating excellent performance when predicting 1–, 3–, and 5–year OS.

### Correlation of clinical characteristics with the risk model

**Fig. 9:**
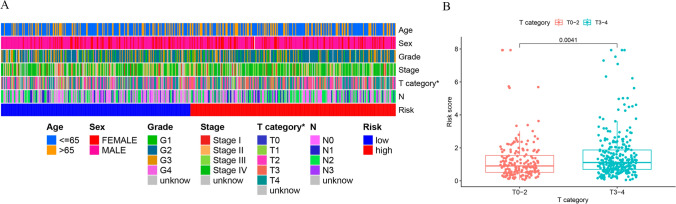
Correlation between clinical characteristics and the risk model. **A** Strip chart of clinical characteristics and risk scores. **B** Scatter diagram illustrating the correlation of T category with risk scores

The association between the clinical characteristics and the risk score was explored. As presented in Fig. 9A, the T category was shown to have a significant link to the risk score. Specifically, the Wilcoxon signed-rank test revealed higher risk scores of HNSCC patients who were at the advanced T category in comparison with those who were at the early T category (Fig. 9B).

### Functional enrichment analysis between HRG and LRG

**Fig. 10:**
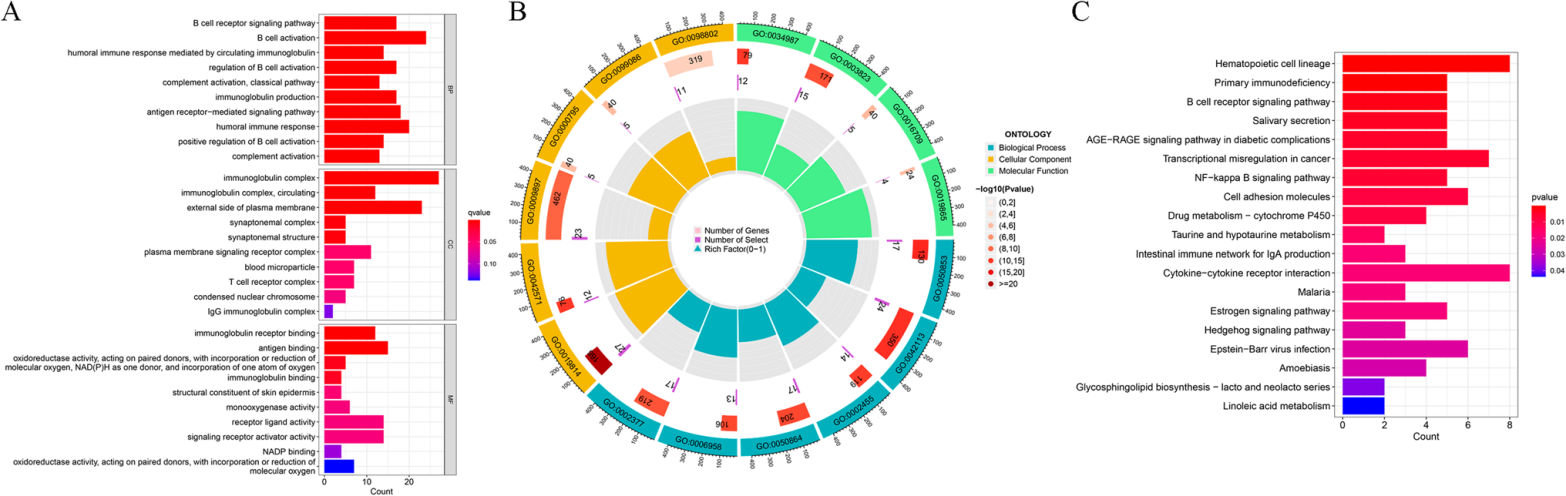
Functional enrichment analysis between low- and high-risk groups. **A**, **B** Bar and circle plots of gene ontology (GO) result. **C** Bar plot of KEGG result

GO analysis was performed on the DEGs between HRG and LRG, which revealed enrichment of the DEGs in immune-related functions encompassing B cell activation, humoral immune response, and immunoglobulin complex (Fig. 10A, B). The KEGG results showed enrichment of these DEGs in pathways like hematopoietic cell lineage, primary immunodeficiency, and B cell receptor signaling (Fig. 10C).

### Immune landscape analysis

**Fig. 11:**
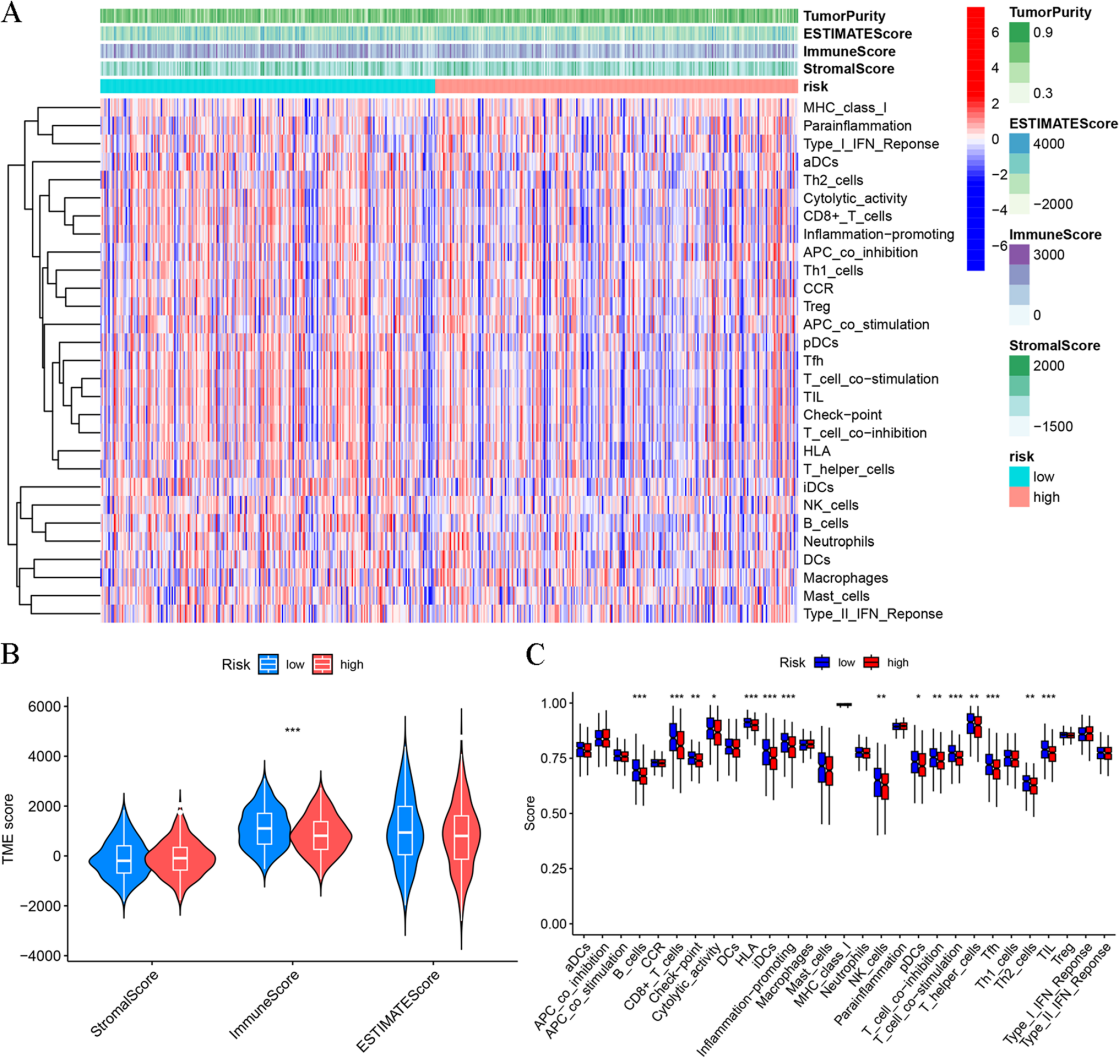
Impact of DRlncRNAs signature on tumor microenvironment in HNSCC. **A** Heatmap showing the link between risk scores and the tumor immune microenvironment. **B** ImmuneScore, StromalScore, and ESTIMATEScore between both risk groups. **C** Violin plot of various infiltration levels of immune cells and immune-related functions between both risk groups

**Fig. 12:**
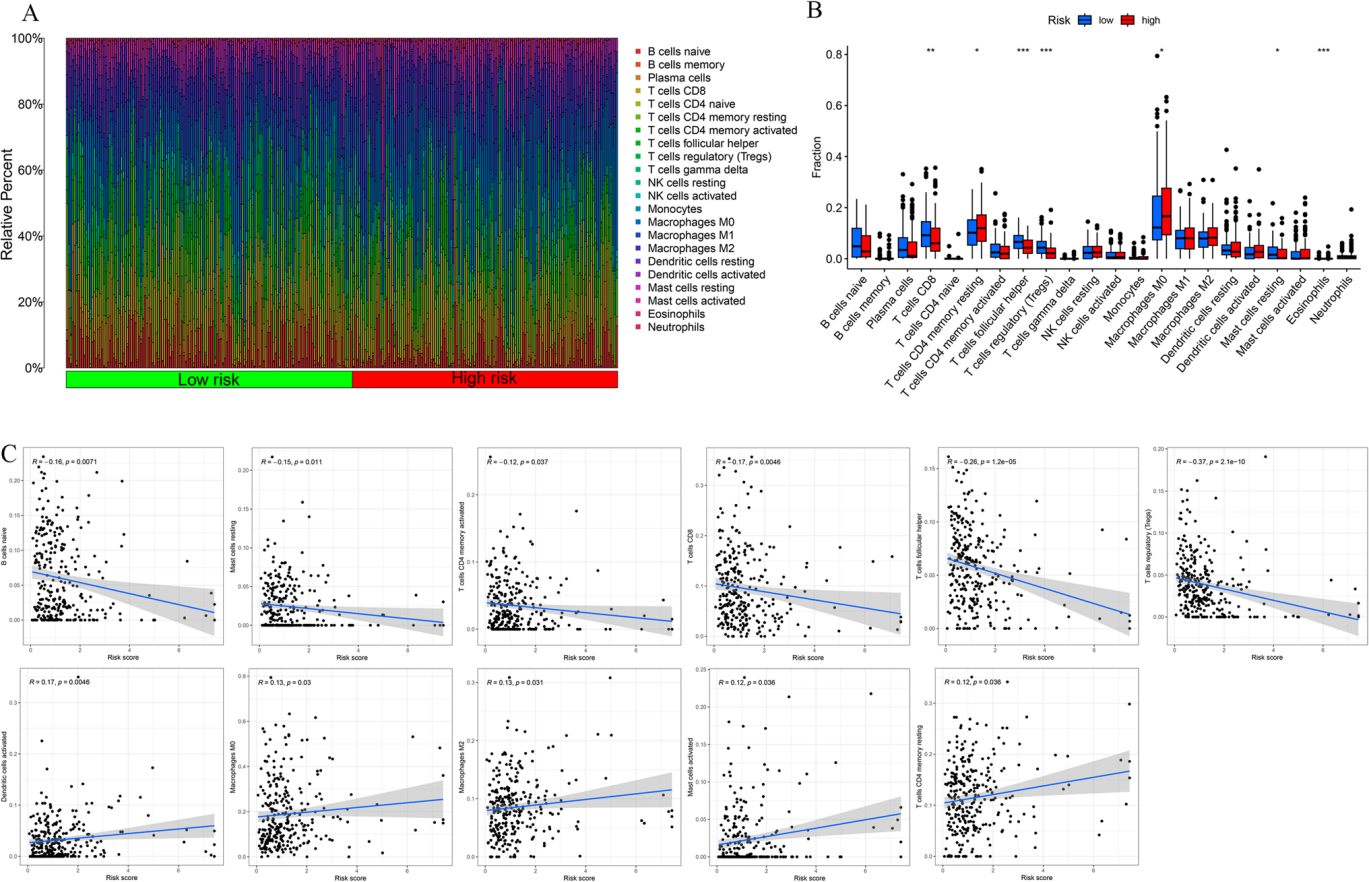
Effect of DRlncRNAs signature on immune cell infiltration. **A** Heatmap of correlation between the risk scores and the tumor-immune cell infiltration. **B** Violin plot of the diverse infiltration levels of immune cells between low- and high-risk groups. **C** Association of risk score with immune cell infiltration

The HRG and LRG were subjected to various analyses to assess the tumor purity, StromalScore, ESTIMATEScore, ImmuneScore, and ssGSEA score of immune-associated function. The variance across the two groups concerning these analyses was illustrated through a heatmap (Fig. 11A). The "ESTIMATE" analysis suggested that patients in the LRG potentially had a higher ImmuneScore than those in the HRG (Fig. 11B). Subsequently, a higher degree of immune-related functions was found to be associated with the individuals with lower risk probably when ssGSEA was applied for assessing the immune cell and immune functions. These functions included checkpoint, inflammation-promoting, HLA, cytolytic activity, as well as T-cell co-stimulation and co-inhibition activities (Fig. 11C). Additionally, the relationship of the risk scores with the tumor-immune cell infiltration was examined, and the resulting data are shown as a heatmap in Fig. 12A. The resulting data of the differential analysis of the infiltration of immune cells between HRG and LRG indicated that individuals in the LRG may showed elevated infiltration levels of T cells follicular helper, T cells CD8, Mast cells resting, and T cells regulatory. Whereas patients with higher risk may be correlated with T cells CD4 memory resting and Macrophages M0 (Fig. 12B). Furthermore, correlation analysis depicted a positive correlation of the risk score with Dendritic cells activated, Macrophages M0, Mast cells activated, Macrophages M2, and T cells CD4 memory resting. Whereas an inverse correlation was noted with B cells naïve, Mast cells resting, T cells CD4 memory activated, T cells CD8, T cells follicular helper, and T cells regulatory (Fig. 12C). These results suggested that the tumor tissues in the LRG may have a higher immune infiltration degree.

### Relationship of risk models with immunotherapy in the HNSCC

**Fig. 13:**
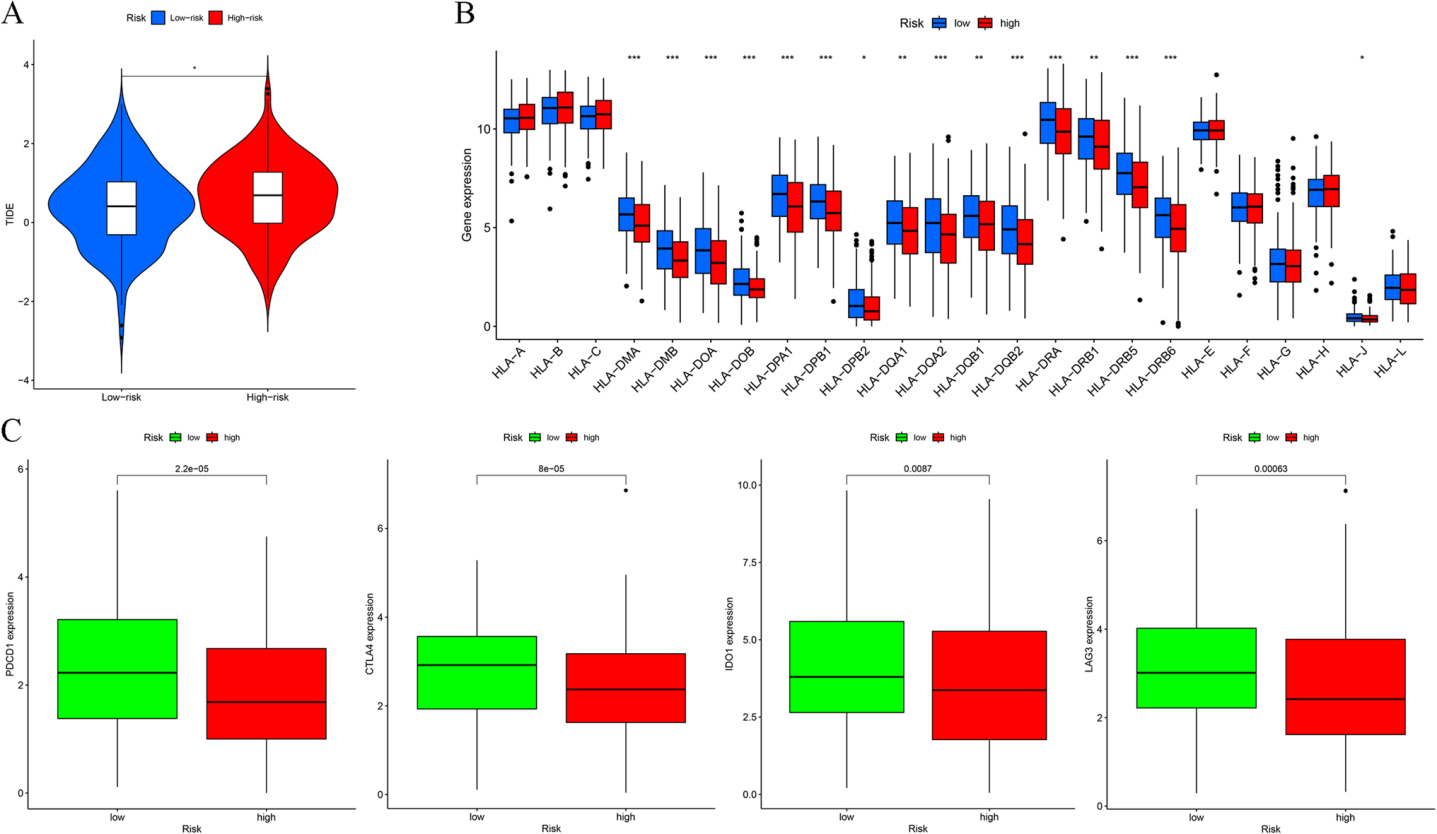
Prediction of immunotherapy response of DRlncRNAs signature to HNSCC. **A** Comparison of Tumor Immune Dysfunction and Exclusion scores between both risk groups (low and high). **B** Comparative expression of human leukocyte antigen (HLA)-related genes in two groups. **C** Comparative assessment of the expression of genes associated with immune checkpoint inhibitors in two groups

The TIDE score, a reflection of the response to immunotherapy, was calculated through the TIDE algorithm. As shown in Fig. 13A, TIDE scores were elevated in the HRG, implying poorer immunotherapy outcomes. The HLA-related gene expression was explored between HRG and LRG. Of these 24 HLA-related genes, 16 depicted elevated expression in the LRG than the HRG (Fig. 13B). Further, the relationship between the risk score and ICIs-related gene expression was also assessed. The resulting data indicated increased expression of PDCD1, CTLA4, IDO1, and LAG3 in the LRG, implying that such individuals might exhibit more favorable effectiveness to immunotherapy (Fig. 13C).

## Discussion

Due to the heterogeneous characteristics and complex carcinogenic mechanisms of HNSCC, the most commonly used TNM staging system cannot accurately interpret the prognosis of patients [27, 28]. Thus, it is crucial to investigate novel biomarkers for HNSCC. Increasing evidence has shown that the stability and tissue-specificity of lncRNAs make them ideal prognostic indicators for tumors [29, 30]. Moreover, the discovery of disulfidptosis has ushered in a new era in antitumor treatment, unveiling a previously unexplored domain of research [10]. This discovery has intriguied interest and investigations into innovative therapeutic approaches for tackling tumors. Herein, a novel DRlncRNA signature was developed for prognostic and immune microenvironment prediction of HNSCC.

In this study, we utilized the TCGA-HNSCC dataset to identify 10 prognosis-linked DRlncRNAs to constitute a novel prognostic disulfidptosis-related lncRNAs signature using LASSO and Cox regression analyses in the training cohort. The risk score derived from this signature negatively correlated with the OS of individuals with HNSCC in the training and the test cohorts. Additionally, it was noted to function as an independent risk indicator on the basis of the KM analysis and multivariate Cox regression. Moreover, PCA, utilized to validate the performance of the model, indicated the discriminative capacity of the lncRNAs in the DRlncRNA signature across patients from different risk groups. Furthermore, the prognostic value of the signature was comparatively assessed with diverse conventional prognostic factors (sex, age, grade, and stage) using ROC curves and C-index value. These comparisons highlighted the superior predictive capacity of the risk model. The clinical subgroup comparisons of age, sex, grade, and stage showed the effectiveness of the prognostic signature in all subgroups, further highlighting the universal applicability of the current risk model. Furthermore, a comparison of this signature with other published HNSCC-related prognosis signatures was executed through time-dependent ROC curves and C-index value. The resulting data implied that our signature was superior to the others in terms of prognosis prediction.

Previous research has established a correlation between TMB and tumor cell behaviors and immunological response [31, 32]. It was noted that a higher TMB is indicative of a worse prognosis for individuals with HNSCC [33, 34]. The survival results of this research were consistent with these reports. TP53, a well-recognized tumor suppressor, influences apoptosis and inhibits proliferation in tumor cells [35]. Ample evidence has previously indicated that TP53 mutation is associated with a worse prognosis in HNSCC [36, 37]. In this study, a high mutation rate of TP53 was found in HNSCC cases, especially in the HRG. Notably, it was found that the HRG demonstrated worse OS irrespective of the TMB level, highlighting the accuracy of our risk model concerning predictive values for HNSCC. In addition, nomograms have been applied in prognosis prediction in various human malignancies [38, 39]. Therefore, a nomogram, based on the clinical features and risk score, was established to accurately predict survival in individuals with HNSCC. The respective AUC values for 1–, 3–, and 5–year OS were 0.787, 0.839, and 0.800 for the nomogram, showing that the nomogram model achieved good predictive accuracy. These collective results show the robustness and efficacy of the model for the prognosis prediction of individuals with HNSCC.

The functional enrichment analysis revealed that the DEGs between HRG and LRG were primarily associated with immune-related biological pathways and processes. It has been well recognized that the TME, particularly the immune microenvironment (IME), is a vital component of tumor biology [40, 41]. Moreover, it was noted that lncRNAs assume an essential role in regulating the tumor IME [42, 43]. Thus, the link between risk scores and IME was explored. Specifically, in terms of the infiltration of immune cells, individuals with lower risk depicted a higher immune score in comparison with the HRG, indicating a greater degree of infiltration. Additionally, ssGSEA indicated increased activation of immune functions in the LRG in comparison with the HRG, confirming the stronger antitumor immune activity in the lower-risk individuals. The study further performed the analysis of immune cell infiltration utilizing CIBERSORT and the ssGSEA methods. Prior research has reported that CD8 T cells, essential for adaptive immunity, confer crucial functions in antitumor immune responses [44–46]. The resulting data were indicative of the elevated infiltration of the CD8 T cells in the individuals at low risk. Furthermore, Xu et al. reported that T follicular helper cells, which have antitumor functions, were associated with satisfactory HNSCC survival [47]. Here, more infiltration of T follicular helper cells was noted in the LRG. Meanwhile, correlation analysis indicated that the risk score had a positive correlation with activated dendritic cells, macrophages M2, macrophages M0, activated mast cells, and resting memory CD4 T cells. It has been reported that macrophages M2, a type of immune suppressive cells, are closely linked to angiogenesis and tissue remodeling. These characteristics contribute to the augmentation of tumor-induced immune suppression, thereby facilitating the progression of tumors [48]. Mast cells, akin to macrophages M2 cells, are frequently recognized as cells that promote tumor growth [49–51]. Concerning the above results, it is reasonable to conclude that disulfidptosis goes along with the modulating of the composition of the IME of the tumor. Additionally, individuals in the HRG may have an attenuated antitumor immune status and worse immunotherapeutic response, which may further lead to poorer prognosis.

There are several limitations in this research that should be considered. Primarily, the training cohort and test cohort were sourced exclusively from the TCGA database. The inclusion of external validation cohorts for analysis would enhance the credibility of the findings. Moreover, the potential relevance of DRlncRNA mechanisms in immunotherapy against HNSCC was not thoroughly investigated in our study, thus warranting further comprehensive research. Additionally, further evidence is required to substantiate the role of ten DRlncRNAs in HNSCC. Hence, it is imperative to design extensive, multicentered prospective studies and wet experiments to validate our findings in subsequent research endeavors.

## Conclusion

Overall, this research effectively established and verified a DRlncRNA signature with the independent ability to independently predict the OS of individuals with HNSCC. Additionally, compelling evidence was presented to support the correlation between the DRlncRNA signature and the TME, as well as immune cell infiltration in HNSCC. Although further studies are needed to elucidate the predictive ability of the DRlncRNA signature on predicting immunotherapy response, these results suggest that this DRlncRNA signature is expected to be a potential biomarker to predict the prognosis of HNSCC.

### Supplementary Information

Additional file1 (XLSX 36 KB)

Additional file2 (XLS 1332 KB)

## Data Availability

The data that support the findings of this study are openly available from The Cancer Genome Atlas database (https://portal.gdc.cancer.gov/).

## Code availability

The codes that were used in this study are available from the corresponding author on reasonable request.
